# Micropropagation, encapsulation, physiological, and genetic homogeneity assessment in *Casuarina equisetifolia*

**DOI:** 10.3389/fpls.2022.905444

**Published:** 2022-08-18

**Authors:** Zishan Ahmad, Vikas Yadav, Anwar Shahzad, Abolghassem Emamverdian, Muthusamy Ramakrishnan, Yulong Ding

**Affiliations:** ^1^Co-Innovation Centre for Sustainable Forestry in Southern China, Nanjing Forestry University, Nanjing, China; ^2^Bamboo Research Institute, Nanjing Forestry University, Nanjing, China; ^3^Plant Biotechnology Section, Department of Botany, Aligarh Muslim University, Aligarh, India

**Keywords:** *Casuarina equisetifolia*, genetic fidelity assessment, micropropagation acclimatization, physiological assessment, synthetic seed production

## Abstract

*Casuarina equisetifolia* is an important tree of the forest, cultivated in tropical and subtropical regions, providing fuelwood, land reclamation, dune stabilization, paper production, and nitrogen fixation. We have developed a systematic *in vitro* propagation protocol in *C. equisetifolia* using nodal segments (NS). Murashige and Skoog (MS) medium augmented with BA (5.0 μM) and NAA (0.5 μM) gave rise to a maximum of 32.00 ± 0.31 shoots per explant (S/E) with shoot length (SL) of 3.94 ± 0.02 cm, and a maximum of 70% regeneration potential (RP) was recorded after 8 weeks of post inoculation. For root induction, *in vitro* derived shoots were transferred to the nutrient medium consisting of a half-strength (½) MS medium augmented with 2.5 μM NAA, which produced a maximum of 12.68 ± 0.33 roots/shoot (R/S) with 3.04 ± 0.50 cm root length (RL) in 60% of culture after 6 weeks. Micropropagated plants with healthy shoots and roots were successfully acclimatized in vermicompost + garden soil + sand (1:2:1) and a maximum survival percentage of 95.1% was recorded. NS was taken from a 6-weeks-old *in vitro* derived plant of *C. equisetifolia* for synthetic seed production, and it was reported that CaCl_2_ · 2H_2_O (100 mM) + Na_2_-alginate (4%) resulted in clear and uniform beads. Furthermore, the maximum conversion of synthetic seeds into plantlets occurred over a period of 4 weeks of storage at 4°C. Scanning Electron Microscopy (SEM) revealed the formation of direct shoot buds without any intermediate callus formation. In addition, the chlorophyll and carotenoid contents of the direct regenerated and mother plant were compared. Similarly, RAPD and ISSR primers were used for genetic homogeneity assessment of the direct regenerated plants, where a total of 18 and 19, respectively, clear and reproducible bands with 100% monomorphism were recorded. The developed micropropagation protocol can certainly be used for large-scale multiplication and germplasm preservation of *C. equisetifolia*. It will also help in meeting the growing demands of *C. equisetifolia* in the forest industry.

## Introduction

*Casuarina equisetifolia* belongs to the family Casuarinaceae, a multipurpose plant, which plays a significant role in economic and environmental refinement, and is widely cultivated in tropical and subtropical areas (Zhang et al., 2008; Zhong et al., 2010). Being a non-leguminous plant, it is capable of fixing nitrogen, worthy for coastal windbreak and sand fixation, ecosystem rehabilitation, soil stabilization, salt resistance, typhoon resistance, afforestation in arid areas, and providing fuelwood, paper, timber, pulp, and charcoal (Pinyopusarerk and Williams, 2000; Schwencke and Caru, 2001; Lui et al., 2003; Dawson, 2008; Zhong et al., 2010; Sayed, 2011; Potgieter et al., 2014). In addition, the plant is also used for its medicinal value due to the presence of several active biological constituents, including phenolic compounds and tannins (Prakash et al., 2007; Ogunwande et al., 2011). The plant has been extensively exploited due to its high demands, especially in the paper and wood industry. Casuarinas are commonly propagated through seeds and seeds of many *Casuarina* species are recalcitrant, have poor germination rates, and exhibit growth variations (Turnbull and Martensz, 1982; Boland et al., 1996). The poor germination rate of seeds is due to insect damage, barriers imposed by seed coat, and empty seeds in seed lots (Sivakumar et al., 2007). For the purpose of commercial exploitation of a genetically stable superior clone and tree breeding programs, vegetative propagation becomes an essential step for selected clones. However, available conventional vegetative propagation methods are lacking due to low yield; hence, *in vitro* propagation methods become the only approach for mass production.

*In vitro* propagation of tree plants is a difficult process for several reasons, *viz*., excessive secretions of phenolic, high rate of contamination, callus formation at the basal end of the explant, and difficulty in the rooting process (Harada and Murai, 1996; Giri et al., 2004). A very limited number of studies are available on the micropropagation of *C. equisetifolia* (Lin et al., 2012; Satheeshkumar and Gupta, 2012; Ren et al., 2022). However, the available methods are not suitable for mass production because of the limited rate of shoot multiplication and poor rooting of the microshoots of *C. equisetifolia*. Considering the above scenario, an advanced *in vitro* mass propagation protocol has been developed for direct organogenesis in *C. equisetifolia*. Maintaining a year-round aseptic environment can also prevent any kind of seed- or soil-borne pathogen attack and this is the reason synthetic seed technology was developed in *C. equistefolia*. Alginate encapsulation opens the door to a cumulative approach for clonal multiplication, storage, transport, and low-cost production and maintenance of elite genotypes, and the species with recalcitrant seeds (Sharma et al., 2013). There are no reports available on the synthetic seed technology in *C. equisetifolia*. Considering the economic and environmental importance and lack of efficient protocol for multiplication, germplasm storage, and transport in *C. equisetifolia*, this study was planned to (1) develop an effective and rapid *in vitro* multiplication protocol for *C. equisetifolia*, (2) synthetic seed production, (3) SEM studies to understand the shoot bud growth, (4) and physiological and genetic homogeneity assessment.

## Methodology

### Explant preparation and disinfection

The nodal segment was taken from a mature tree of *C. equisetifolia* planted on the Aligarh Muslim University campus. Washing of NS was carried out for 30 min with normal tap water followed by treatment with Teepol (5%, v/v) for 15 min and again washing with running tap water for 15 min. Surface sterilization was performed with mercuric chloride (HgCl_2_, 0.1% w/v) for 5 min followed by washing with autoclaved double distilled water (three times) before inoculation.

### Nutrient media and culture conditions

Murashige and Skoog (1962) was used as a nutrient medium supplemented with 3% (w/v) sucrose, optimized plant growth regulators (PGRs), adjusted to pH 5.8 with 1 N NaOH/HCl before gelling with 0.8 % (w/v) agar. The media was autoclaved at 121°C at 1.06 kg cm^−2^ for 15–20 min. The nutrient medium (15–20 ml) was allocated in the culture tube (25 × 150 mm) and flask (50 ml in 100 ml flask). Culture vessels were placed in a culture room at 25 ± 2°C under a 16-h light/8-h dark photoperiod. Woody Plant Medium (WPM) (McCown and Lloyd, 1981) and B5 medium (Gamborg et al., 1968) were also used with optimized PGRs concentration for comparative studies of nutrient analysis.

### Establishment of *in vitro* culture

One explant was inoculated into each culture tube containing a nutrient medium. MS basal medium augmented with cytokinins like 6-benzyladenin (BA), Metatopoline (mT), Kinetin (Kn), 2-and isopentanyladenin (2-iP) at different concentrations, *viz*., 1.0, 2.5, 5.0, 7.5, and 10.0 μM and auxins, *viz*., α-naphthalene acetic acid (NAA), indole-3-acetic acid (IAA), and indole-3-butyric acid (IBA) at different concentrations, *viz*., 0.1, 0.5, and 1.0 μM, were used. Shoot induction data were noted after 6–8 weeks of culture. Subculturing of regenerating shoots was carried out on an optimized medium up to eight stages and 4 weeks of interval. The optimized PGR concentration was used for comparative nutrient analysis as stated in Section Nutrient media and culture conditions.

### Synthetic seed production and storage

Encapsulation of the nodal segment obtained from *in vitro* established (6 weeks old) cultures of *C. equisetifolia* was performed with Na_2_-alginate (4%) solution and left for 25 min by dropping them into CaCl_2_ · 2H_2_O (100 mM) solution. These were then rinsed with sterile liquid MS medium and transferred to a flask containing optimum concentration of germination media and then placed in a culture room while the data for the %germination frequency of encapsulated beads was recorded after 6 weeks. Encapsulated NS having MS or DDW gel matrix and non-encapsulated nodal segments were kept in sterilized beakers sealed with two layers of Para Film and stored at 4°C. Five various exposure times, *viz*., 1, 2, 4, 6, and 8 weeks were assessed for synseed regeneration, conversion into plantlets was evaluated after each storage period, and data were collected after 6 weeks of culture.

### *In vitro* rooting and acclimatization

Rooting of micropropagated shoots (both direct and synseed derived) was carried out on full- and half-strength MS medium supplemented with different auxins, *viz*., NAA, IAA, and IBA at concentrations of 1.0, 2.5, and 5.0 μM, and data were noted after 6 weeks. Acclimatization of healthy plantlets were carried out in thermocol cups full of three different kinds of substrates, *viz*., autoclaved vermicompost:garden soil:sand (1:2:1), soilrite, and vermicompost. Then, well-acclimatized plantlets were moved to earthen pots after 4 weeks, having soil + green manure in the ratio of 2:1, and initially left it in the culture room for 4 weeks. After that the earthen pots were moved to field conditions.

### Chlorophyll and carotenoid assessment

Fresh leaves (100 mg) taken from direct regenerated plants were grinded in 5 ml acetone (80%) with the help of a mortar and pestle and Whatman filter paper was used to filter out the suspension. The final volume of the filtrate was prepared to 10 ml with acetone (5 ml). To calculate the chlorophyll and carotenoid contents, the optical density (O.D.) was recorded at 645 and 663 nm and 480 and 510 nm, respectively, with the help of a spectrophotometer (UV-Pharma Spec 1700, Shimadzu, Japan).

### Scanning electron microscopy (SEM) examination

A 3-week-old nodal segment culture showing multiple shoot buds was taken for SEM analysis. A 2% buffered gluteraldehyde was used to fix the sample and left at 4°C overnight, followed by rinsing in 0.1 M sodium phosphate buffer at pH 7.0. Then, the sample was dehydrated in an ascending acetone series from 30 to 90%, and drying with carbon was performed at a critical point. Furthermore, it was mounted on aluminum stubs with silver paint and coated with gold followed by Scanning Electron Microscope (SEM, 501B, Phillips, Holland) at 10 KV and images were captured digitally.

### Genetic homogeneity assessment

Cetylmethylammonium bromide (CTAB) method (Doyle and Doyle, 1990) was used for the extraction of DNA from nine direct regenerated plants of *C. equisetifolia*, followed by screening of extracted DNA on UV–vis spectrophotometer for clarity. A set of ISSR (UBC, Vancouver, BC, Canada) and RAPD (kit OPL) primers were used for polymerase chain reaction (PCR) on a thermocycler (Biometra, Germany). The PCR reaction mixture (20 μl) was prepared with 10X buffer (2 μl) + 10 mM dNTPs (0.4 μl) + 25 mM MgCl2 (1.2 μl) + 3 Unit Taq polymerase (0.2 μl) + 10 μM primers + 40 ng template DNA. The condition of PCR was set as follows: 45 cycles, 94°C denaturation step (5 min, no repeats), annealing temperature (55°C, 1 min), elongation step (72°C, 1 min), and final extension (72°C, 10 min). The separation of amplified product was performed on electrophoresis in 0.8% (w/v) agarose gels with 1 μg/mL ethidium bromide in TAE buffer (pH 8.0) run at 50 V for 2 h and visualized on a UV trans-illuminator (BIO RAD, Hercules, CA, USA). The size of the amplified DNA was evaluated using a ladder DNA marker (New England Biolabs, UK, 50 ng/μl, 1kb).

### Statistical analysis

All experiments were performed with 10 replicates and repeated three times. The data were analyzed statistically by one-way *ANOVA* and represented as mean ± standard error (SE). Duncan's multiple-range test (at *P* ≤ 0.05) was used to indicate the significant difference.

## Results

### Effect of season on the regeneration efficiency of the explant

Plant material was collected throughout the year and inoculated on BA supplemented medium. The explant responded maximum if collected in the month of April and 71.21 ± 0.04% out of 82.13 ± 0.21% of the established sterile culture witnessed axillary bud sprouting (Table 1). Whereas, the least bud sprouting was observed in the months of June to October. A minimal response was recorded in the month of June, 4.21 ± 0.21% of the culture responsive out of 9.10 ± 1.21% survived sterile cultures. The explant collected during April was selected for *in vitro* study in *C. equisetifolia*.

**Table 1:** Response of explant against various seasons (after 15 days of post inoculation).

**Months**	**Sterile** **(%)**	**Sterile** **sprouted** **(%)**	**Contaminated (%)**
January	40.12 ± 1.02^b^	28.50 ± 1.11^c^	58.56 ± 1.11^ab^
February	41.30 ± 1.22^b^	29.10 ± 1.02^c^	59.30 ± 1.01^ab^
March	78.85 ± 0.31^a^	69.31 ± 0.12^ab^	18.12 ± 0.10^a^
April	82.13 ± 0.21^a^	71.21 ± 0.04^ab^	17.25 ± 0.04^cd^
May	80.21 ± 0.11^a^	69.91 ± 0.01^ab^	19.10 ± 0.12^c^
June	9.10 ± 1.21^d^	4.21 ± 0.21^d^	91.2 ± 0.13^a^
July	9.32 ± 1.11^d^	4.50 ± 0.30^d^	91.5 ± 0.12^a^
August	9.80 ± 1.22^d^	4.70 ± 0.40^d^	91.8 ± 0.13^a^
September	10.01 ± 1.21^d^	5.01 ± 0.40^d^	90.1 ± 0.14^a^
October	10.21 ± 1.22^d^	5.10 ± 0.61^d^	89.2 ± 0.12^ab^
November	38.80 ± 1.14^a^	25.20 ± 1.12^a^	62.10 ± 1.11^ab^
December	39.32 ± 1.23^a^	26.30 ± 1.21^a^	60.36 ± 1.21^ab^

Different letters in the same column are significantly different at P ≤ 0.05 [Duncan's multiple range test (DMRT)].

### Direct organogenesis, shoot formation, and proliferation

**Figure 1:**
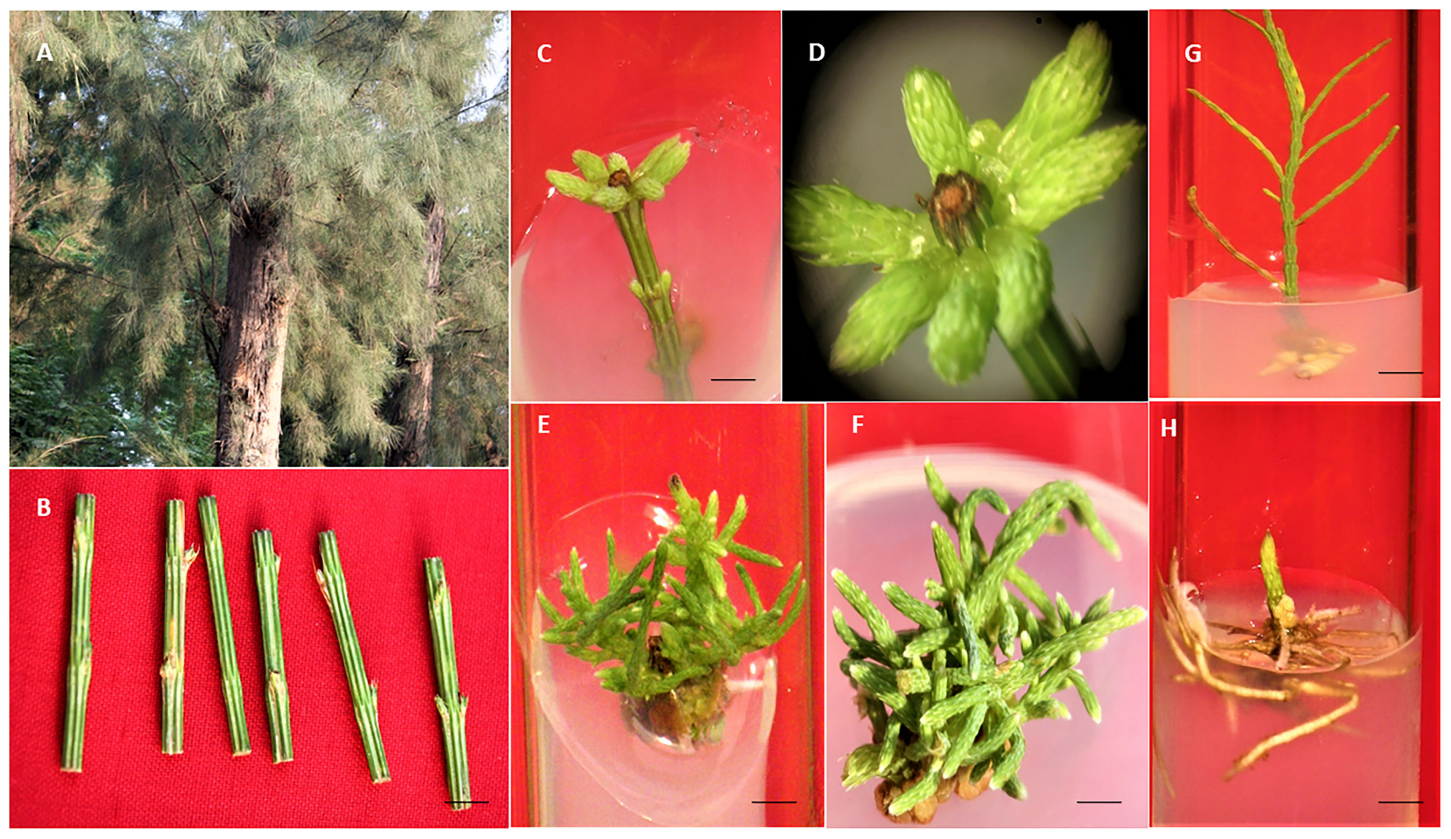
*In vitro* propagation protocol in *C. equisetifolia*. **(A)** Mother plant. **(B)** NS (Bar = 0.72 cm). **(C)** Shoot induction on MS + BA (5.0 μM) (3 weeks old) (Bar = 0.58 cm). **(D)** Close view of culture A (Bar = 0.58 cm). **(E)** Multiple shoots on MS + BA (5.0 μM) (8 weeks old) (Bar = 0.61 cm). **(F)** Multiple shoots on MS + BA (5.0 μM) + NAA (0.5 μM) (8 weeks old) (Bar = 0.62 cm). **(G)** Root initiation on half strength ½ MS + NAA (2.5 μM) (2 weeks old) (Bar = 0.43 cm). **(H)** multiple roots on ½ MS + NAA (2.5 μM) (6 weeks old) (Bar = 0.21 cm).

When nodal segments were inoculated on MS basal medium (control) no shoot regeneration was observed and the explant remained green and, therefore augmentation of PGR was required for axillary bud sprouting. Five cytokinins, i.e., BA, mT, Kn, and 2iP at 0.1, 0.25, 0.5, 1.0, 2.5, 5.0, 7.5 and 10.0 μM concentration were applied (Table 2). Swelling followed by shoot buds emergence to the nodal segment was observed within 18 days of inoculation, and after 25 days of inoculation, the differentiation of shoot buds was observed. Among all cytokinins, BA was found to be optimum, and a treatment consisting of MS + BA (10.0 μM) produced 23.90 ± 0.40 shoots/explant (S/E) having shoot length (SL) of 1.72 ± 0.07 cm with 70% regeneration potential (RP) in 8 weeks (Figures 1A–E). Increasing the concentration of BA beyond the optimum level resulted in a decrease in %RP and shoots formation. Moreover, in comparison to the other three cytokinins, mT was found more effective than Kn and 2-iP. However, lower concentration of mT (2.5 μM) was more effective in contrast to BA, Kn, and 2-iP, and a maximum of 16.80 ± 0.58 S/E and SL of 1.46 ± 0.05 cm with a RP of 60% was recorded after 8 weeks of culture. Similarly, at higher concentration (7.5 μM), Kn and 2-iP produce 11.14 ± 0.35 S/E and 6.72 ± 0.21 S/E and SL was 1.10 ± 0.20 cm and 1.36 ± 0.09 cm, respectively, in 60% of cultures, after 8 weeks of inoculation (Table 2). For further improvement in multiple shoots differentiation, combinations of optimal cytokinin and different auxins, *viz.*, NAA, IBA, and IAA at concentrations of 0.1, 0.5, and 1.0 μM were tried (Table 3). MS + BA (5.0 μM) + NAA (0.5 μM) treatment gave rise to a maximum of 32.00 ± 0.31 S/E and SL of 3.94 ± 0.02 cm was recorded after 8 weeks post-inoculation along with 70% of RP (Figure 1F). NAA at higher concentration (1.0 μM) resulted in decreased S/E (22.60 ± 0.24) and RP (50%) with heavy callus formation leading to the inhibition of growth. Furthermore, IBA was more effective than IAA, and a treatment consisting of MS + BA (5.0 μM) + IBA (0.1 μM) gave rise to 20.60 ± 0.24 S/E with SL of 3.72 ± 0.03 cm after 8 weeks of post-inoculation with 60% RP. While, 19.80 ± 0.20 S/E with SL of 3.40 ± 0.03 cm recorded at 8 weeks post-inoculation with 60% RP on MS + BA (5.0 μM) + IBA (0.1 μM) treatment (Table 3).

**Table 2:** Direct shoot regeneration (means ± SE) from NS inoculated on different cytokinins (after 8 weeks of culture).

**PGR** **(**μ**M)**				**Regeneration (%)**	**Mean no. of shoots/explant**	**Mean shoot length (cm)**
**BA**	**mT**	**Kn**	**2ip**			
Control				00	0.00 ± 0.00^l^	0.00 ± 0. 00^h^
1.0	–	–	–	30	8.94 ± 0.33^e^	1.24 ± 0.06^cde^
2.5	–	–	–	40	12.36 ± 0.30^c^	1.56 ± 0.06^ab^
5.0	–	–	–	70	23.90 ± 0.40^a^	1.72 ± 0.07^a^
7.5	–	–	–	50	15.80 ± 0.73^b^	1.38 ± 0.03^bcd^
10.0	–	–	–	40	11.68 ± 0.56^cd^	1.24 ± 0.05^cde^
–	1.0	–	–	40	9.64 ± 0.51^de^	1.32 ± 0.05^cde^
–	2.5	–	–	60	16.80 ± 0.58^b^	1.46 ± 0.05^bc^
–	5.0	–	–	30	11.52 ± 0.13^cd^	1.38 ± 0.03^bcd^
–	7.5	–	–	40	8.70 ± 0.20^def^	1.22 ± 0.05^de^
–	10.0	–	–	50	7.76 ± 0.34^fg^	1.24 ± 0.06^cde^
–	–	1.0	–	40	3.38 ± 0.18^k^	0.82 ± 0.05^g^
–	–	2.5	–	40	5.64 ± 0.18^ij^	0.90 ± 0.03^fg^
–	–	5.0	–	50	7.42 ± 0.47^g^	0.82 ± 0.05^g^
–	–	7.5	–	60	11.14 ± 0.35^d^	1.10 ± 0.20^ef^
–	–	10.0	–	50	6.20 ± 0.37^hi^	0.94 ± 0.05^fg^
–	–	–	1.0	30	2.46 ± 0.21^k^	1.10 ± 0.04^ef^
–	–	–	2.5	40	4.62 ± 0.18^j^	1.24 ± 0.06^cde^
–	–	–	5.0	50	4.78 ± 0.12^j^	1.34 ± 0.05^bcd^
–	–	–	7.5	60	6.72 ± 0.21^gh^	1.36 ± 0.09^bcd^
–	–	–	10.0	40	3.10 ± 0.04^k^	1.18 ± 0.06^de^

Different letters in the same column are significantly different at P ≤ 0.05 (DMRT).

**Table 3:** Adventitious microshoots formation (means ± SE) on optimized cytokinin with different auxins (8 weeks after culture).

**Auxin (**μ**M)**			**Regeneration** **(%)**	**Mean no. of shoots/explant**	**Mean shoot length (cm)**
**NAA**	**IBA**	**IAA**			
0.1	–	–	60	23.40 ± 0.50^b^	3.54 ± 0.02^d^
0.5	–	–	70	32.00 ± 0.31^a^	3.94 ± 0.02^a^
1.0	–	–	50	22.60 ± 0.24^b^	3.68 ± 0.02^bc^
–	0.1	–	60	20.60 ± 0.24^b^	3.72 ± 0.03^b^
–	0.5	–	50	19.60 ± 0.24^c^	3.60 ± 0.04^cd^
–	1.0	–	40	15.40 ± 0.24^d^	3.58 ± 0.02^cd^
–	–	0.1	50	10.20 ± 0.20^e^	3.14 ± 0.02^f^
–	–	0.5	40	15.40 ± 0.24^d^	3.32 ± 0.07^e^
–	–	1.0	60	19.80 ± 0.20^c^	3.40 ± 0.03^e^

Different letters in the same column are significantly different at P ≤ 0.05 (DMRT).

### Effect of different nutrient medium and subculture passage

Three basal medium, *viz.*, MS, WPM, and B5 were used with optimized phytohormone combination treatment BA (5.0 μM) + NAA (0.5 μM). Of these, MS medium was found to be best and gave rise to 32.00 ± 0.31 S/E followed by WPM with 23.50 ± 0.58 S/E and B5 with 15.00 ± 0.31 S/E (Supplementary Figure 1). MS basal medium was used throughout the experiment. A continuous (up to sixth subculture passage) subculture of the regenerating tissue was performed on the fresh medium augmented with best-optimized treatments BA (5.0 μM) and NAA (0.5 μM). The healthy shoots simultaneously transferred to the rooting medium, while the remaining tissue was further subcultured in a fresh regeneration medium. The rate of shoot multiplication increased during the first to the fourth subculture recording 42.20 ± 0.82 S/E, thereafter, S/E decreased to 41.20 ± 0.83 after the fifth subculture passage, and remain persistent, followed by a decline in S/E after every subculture along with various abnormalities, *viz*., yellowing of shoots and early leaf fall were observed (Supplementary Figure 2).

### *In vitro* rooting, hardening, and acclimatization

**Figure 2:**
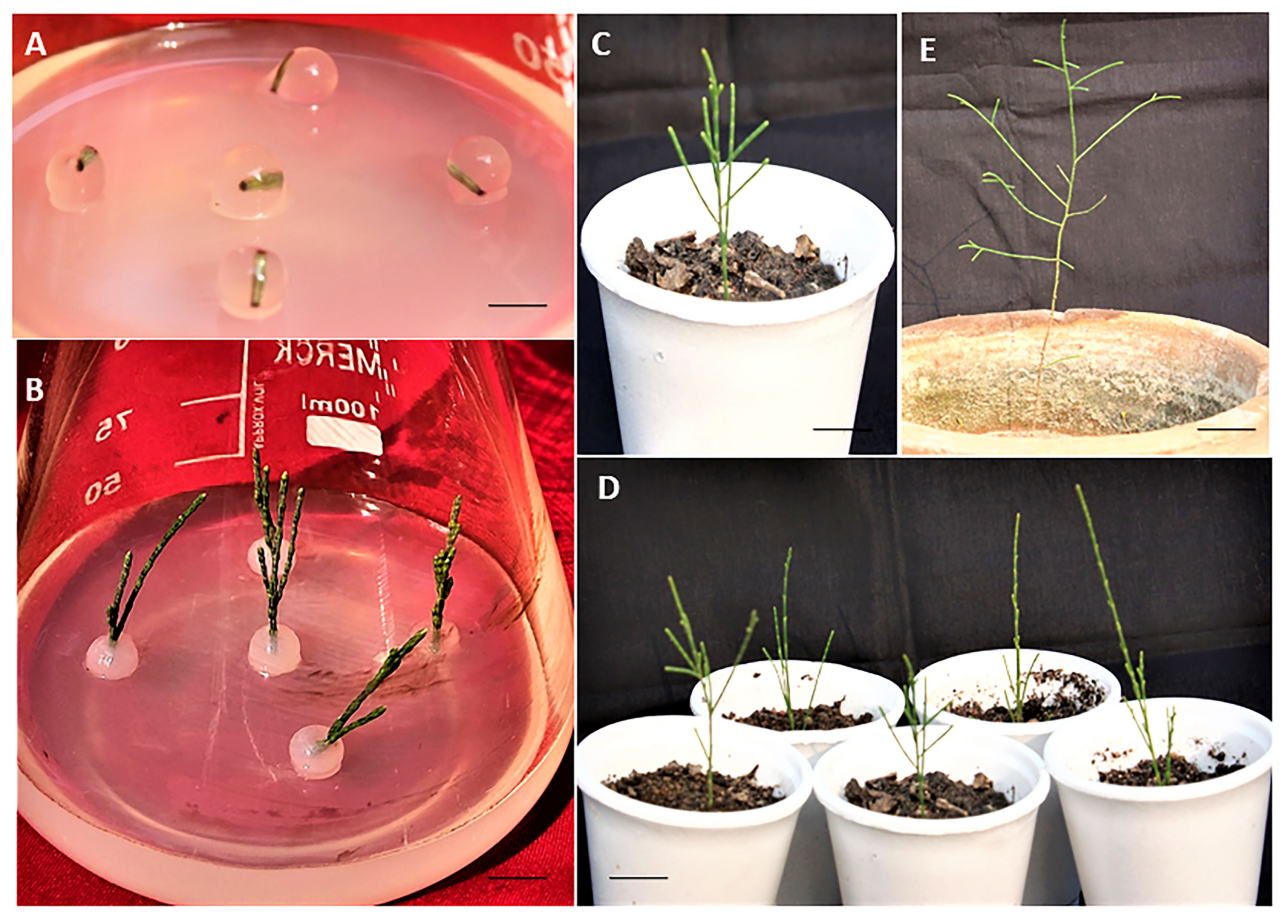
Synthetic seed production and acclimatization of direct regenerated plants of *C. equisetifolia*. **(A)** Uniform synthetic seed on MS + BA (5.0 μM) + NAA (0.5 μM) (1 day old) (Bar = 1.03 cm). **(B)** Sprouting of multiple shoots on the same medium (4 weeks old) (Bar = 0.91 cm). **(C,D)** Successfully acclimatized plant (8 weeks old). (Bar = 1.8 cm and 2.1). **(E)** Transfer in the field (6 months old) (Bar = 7.1 cm).

*In vitro* rooting for microshoots was performed using full- and half-strength MS medium without auxins or augmented with auxins, for example NAA, IBA, and IAA. There was no root induction in full- or half-strength MS medium devoid of any auxin and basal callusing was observed at the cut end. The addition of auxins helps to induce root to the microshoots and a treatment consisting of ½ MS + 2.5 μM NAA was found to be best and gave rise to a maximum of 12.68 ± 0.33 roots/shoot, and a root length of 3.04 ± 0.50 cm was recorded at 6 weeks of post-transfer in 60% microshoots (Table 4; Figures 1G,H). Further increase in the concentration of NAA, i.e., 5.0 μM, brings the overall decrease in rooting to 30%, and only 8.26 ± 0.50 root/shoot was recorded. The other two auxins, *viz.*, IBA and IAA were found to be less effective than NAA, and treatment with ½ MS + 1.0 μM IBA produces only 4.66 ± 0.23 roots/shoot, and root length of 2.5 cm was recorded in 40% of microshoots at 6 weeks of post culture. Similarly, ½ MS + 1.0 μM IAA gave rise to 1.56 ± 0.40 root/shoot and a root length of 2.56 ± 0.02 cm was recorded in 50% of microshoots at 6 weeks post culture. Micropropagated plants with healthy shoot and root were well hardened off in the three types of planting substrates under a controlled environment (Supplementary Figure 3). A combination of vermicompost:garden soil:sand (1:2:1) was found most favorable planting substrate, as 95.1% of plants survived. While in the other two planting substrates, *viz*., vermicompost and soilrite, 80.4% and 70.5% of plant survival were recorded, respectively (Figure 2C). Furthermore, 84.6% of survival was recorded when transferred from a controlled or culture room environment to field conditions. *In vitro*-derived plants grew well and showed no variation in morphological character as compared to the mother plant (**Figure 5D**).

**Table 4:** *In vitro* rooting in microshoots (means ± SE) (after 6 weeks of transfer).

**Treatment (μM)**	**Response (%)**	**Mean no. of roots/shoot**	**Mean root** **length (cm)**
MS	0.00	0.00 ± 0.00^h^	0.00 ± 0.00^d^
½ MS	0.00	0.00 ± 0.00^h^	0.00 ± 0. 00^d^
½ MS + NAA (1.0)	40	9.74 ± 0.22^b^	2.94 ± 0.24^a^
½ MS + NAA (2.5)	60	12.68 ± 0.33^a^	3.04 ± 0.50^a^
½ MS + NAA (5.0)	30	8.26 ± 0.50^c^	2.98 ± 0.37^a^
½ MS + 1BA (1.0)	40	4.66 ± 0.23^d^	2.56 ± 0.02^b^
½ MS + IBA (2.5)	30	2.98 ± 0.23^e^	2.38 ± 0.03^c^
½ MS + IBA (5.0)	30	1.48 ± 0.19^g^	2.36 ± 0.02^c^
½ MS + IAA (1.0)	50	1.56 ± 0.40^g^	2.56 ± 0.02^b^
½ MS + IAA (2.5)	60	3.18 ± 0.03^e^	2.98 ± 0.37^a^
½ MS + IAA (5.0)	60	2.14 ± 0.02^f^	2.36 ± 0.02^c^

Different letters in the same column are significantly different at P ≤ 0.05 (DMRT).

### Synthetic seed production, regeneration, and storage

The concentrations of Na_2_-alginate and CaCl_2_·2H_2_O significantly affect the size and texture of the synthetic seed. In our study, CaCl_2_ · 2H_2_O (100 ml) + Na_2_-alginate (4%) gave rise to clear and uniform synthetic seeds and 68.60 ± 1.86% conversion into plantlets was recorded (Figures 2A,B; Table 5). Two types of synthetic seed germination medium were used, e.g., MS medium devoid of any PGRs as control and PGRs supplemented medium. The control medium exhibits minimal germination of 48.60 ± 1.86% to the synthetic seed, while a maximum germination of 77.0% was recorded on MS + 5.0 μM BA + 0.5 μM NAA and 74.20 ± 1.90% germination on MS + 5.0 μM BA + 0.1 μM NAA (Table 6). Furthermore, a decrease in germination was observed in other cytokinin and auxin supplemented medium (Table 6). Storage of synthetic seeds at low temperature also affected the regeneration frequency of synthetic seeds. In our study, storage at 4°C for a period of 4 weeks was observed, with a maximum conversion of 71% synthetic seeds into plantlets and 3.6 shoots recorded at 8 weeks of post inoculation. Additionally, a decrease in the conversion rate was observed as the storage period increased (Table 7).

**Table 5:** Effect of Na_2_-alginate + CaCl_2_ · 2H_2_O on conversion of encapsulated NS (means ± SE) (8 weeks after culture).

**Sodium alginate (%, w/v)**	**Conversion response** **(%) into plantlets**
1	Fragile beads
2	Fragile beads
3	64.40 ± 1.91^a^
4	68.60 ± 1.86^a^
5	33.60 ± 1.56^b^
**Calcium chloride (mM)**	
25	Fragile beads
50	Fragile beads
75	42.00 ± 0.94^b^ (but soft to handle)
100	68.60 ± 1.86^a^
200	24.40 ± 1.28^c^

Different letters in the same column are significantly different at P ≤ 0.05 (DMRT).

**Table 6:** Conversion response (means ± SE) of encapsulated NS on different treatments (8 weeks after culture).

**Treatments (μM)**	**Conversion response** **(%) into plantlets**
MS	48.60 ± 1.86^e^
MS + BA (5.0) + NAA (0.1)	74.20 ± 1.90^ab^
MS + BA (5.0) + NAA (0.5)	77.00 ± 2.09^a^
MS + mT (2.5) + IBA (0.5)	68.80 ± 1.77^c^
MS + mT (2.5) + IBA (1.0)	62.80 ± 1.85^c^
MS + Kn (7.5) + IBA (0.5)	59.60 ± 1.80^d^

Different letters in the same column are significantly different at P ≤ 0.05 (DMRT).

**Table 7:** Effect of low temperature (4°C) on the conversion of encapsulated NS into plantlets (means ± SE) on best treatment (8 weeks after culture).

**Storage** **periods** **(weeks)**	**Conversion response (%)**	**Mean No.** **of shoots**
0	42.3 ± 0.66^e^	1.4 ± 0.33^c^
1	45.3 ± 0.66^d^	1.8 ± 0.33^c^
2	53.3 ± 0.33^c^	2.3 ± 0.36^b^
3	65.3 ± 0.33^b^	2.7 ± 0.33^b^
4	71.6 ± 0.33^a^	3.6 ± 0.66^a^
5	36.3 ± 0.33^f^	1.0 ± 0.11^c^

Different letters in the same column are significantly different at P ≤ 0.05 (DMRT).

### Chlorophyll, carotenoids assessment, and SEM examination of the cultured nodal segment

**Figure 3:**
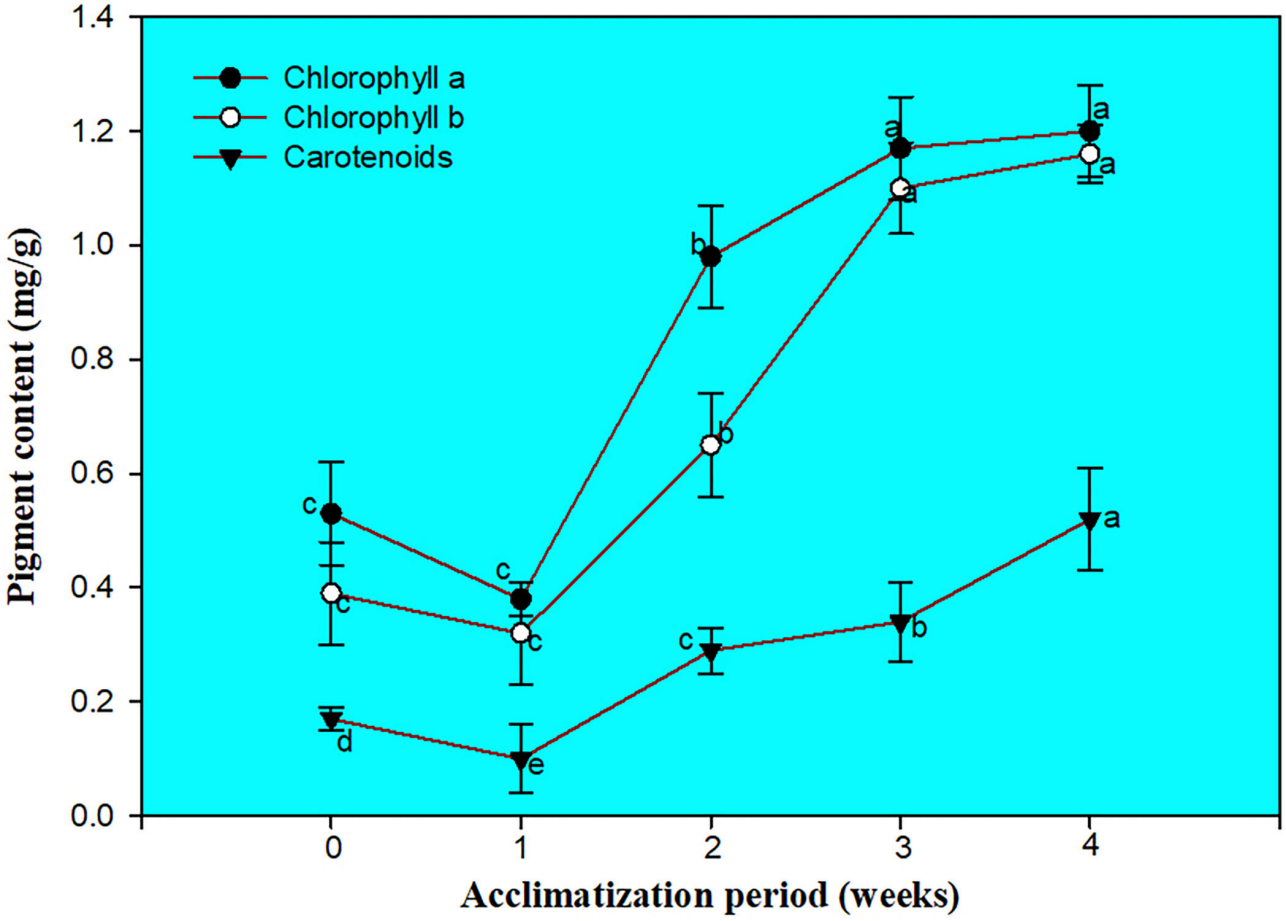
Changes in chlorophyll a and b and carotenoid contents (Mean ± SE).

**Figure 4:**
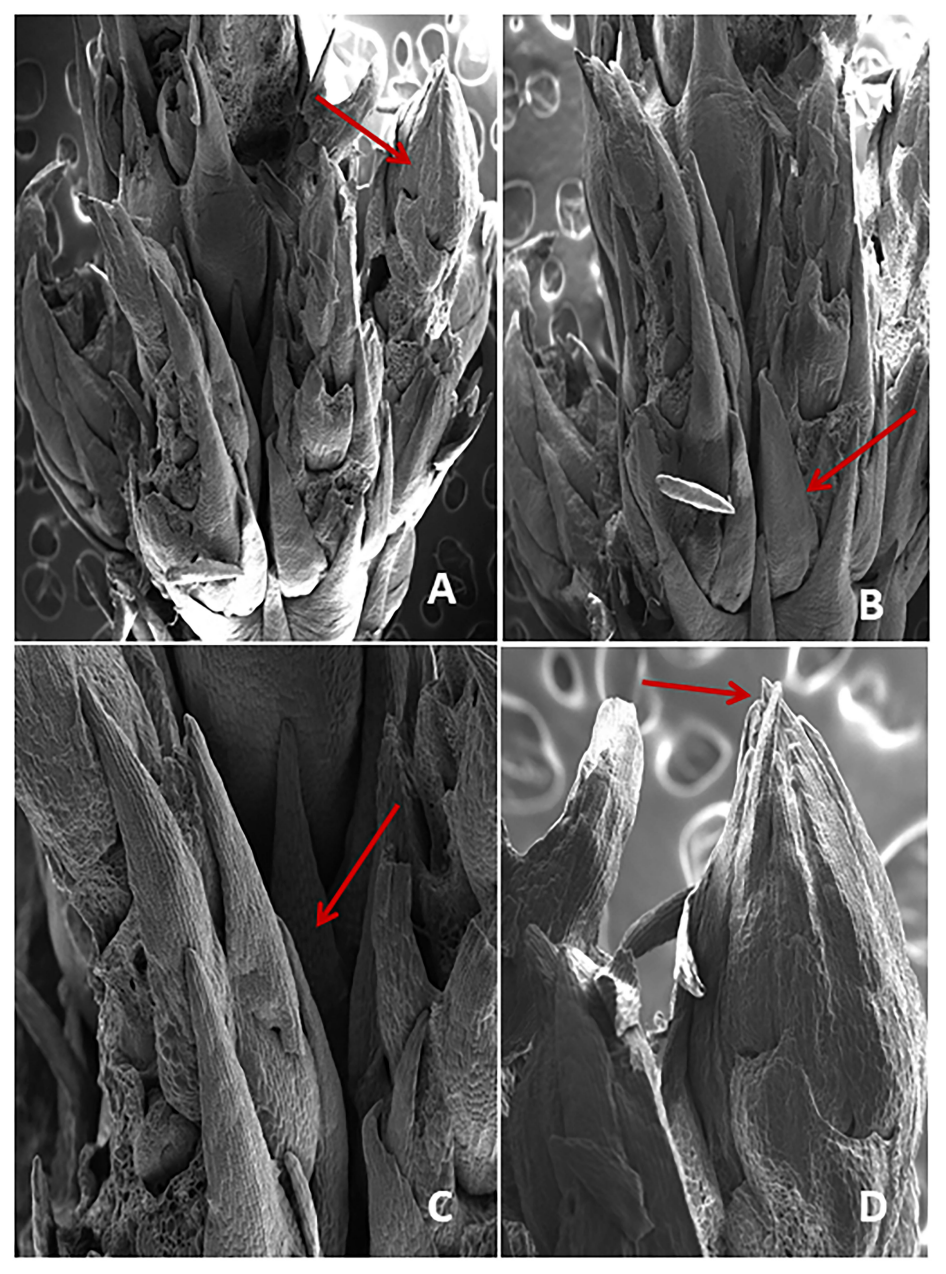
**(A–D)** SEM examination showing the crown of direct shoot bud formation from the nodal region of explant (arrow).

The screening of chlorophyll a, b, and carotenoids were performed at different acclimatization periods of 7, 14, 21, and 28 days. A fall in chlorophyll and carotenoid contents was recorded during the initial days of acclimatization, followed by a gradual increase as the days of acclimatization progressed. The maximum contents of chlorophyll a (1.20 mg/g), b (1.16 mg/g), and carotenoid (0.52 mg/g) were recorded after 4 weeks of acclimatization (Figure 3). SEM examination of cultured NS witnessed the induction of shoot bud in direct mode or without any intervening callus phase and shows multiple shoot buds formation in cluster form and these shoots buds originated from the axil of each scaly leaf. A frontal as well as a posterior view of direct shoot bud emergence from NS shows the integrity of the cluster with explant witnessing the absence of any callus formation (Figure 4).

### Genetic fidelity assessment

**Figure 5:**
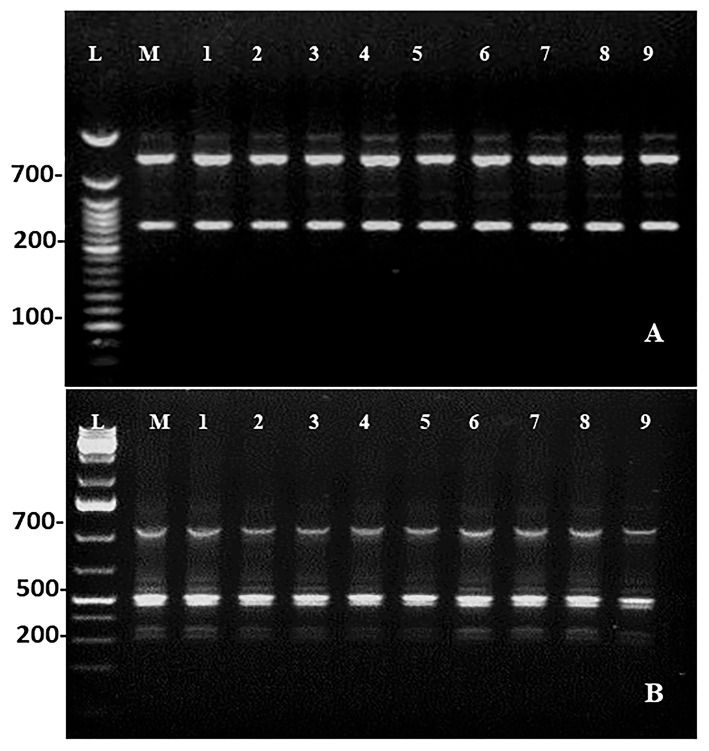
DNA profiles of the mother (Lane M) with DNA ladder (lane L) and micropropagated plants (Lanes 1–9). **(A)** RAPD (OPU-08). **(B)** ISSR (UBC-812).

Maintenance of genetic similarity of *in vitro* derived plants with the mother plants is an important steps of micropropagation. Two molecular markers, *viz*., RAPD (Random Amplified Polymorphic DNA) and ISSR (Inter Simple Sequence Repeats) were used to examine the genetic similarity of direct regenerated and mother plants. The RAPD and ISSR primers produced a total of 18 and 19 bands, respectively. The bands were clear and reproducible with 100% monomorphism. The RAPD marker, OPU-8, gives rise to the highest number of four bands, and the ISSR marker, UBC-812, gives rise to five bands (Table 8; Figure 5).

**Table 8:** List of RAPD and ISSR primers and their sequences along with scorable bands and size range.

**Molecular marker**	**Primer code**	**Primer sequences(5^′^−3^′^)**	**Amplified band**	**Size range (bp)**
RAPD	OPL-01	5′GGCATGACCT3′	1	200–400
	OPL-04	5′GACTGCACAC3	4	300–700
	OPL-06	5′GAGGGAAGAG3′	2	400–1,000
	OPU-05	5′TAGGAGGTGG3′	4	600–1,500
	OPU-08	5′TGCGAGAGAA3′	4	500–2,000
	OPU-10	5′TGACAGAGTT3′	3	300–500
ISSR	UBC-810	(GA)8T	2	500–1,200
	UBC-812	(GA)8A	5	300–2,500
	UBC-830	(AG)8YT	4	1,000–3,500
	UBC-841	(GAA)8	5	200–1,500
	UBC-870	(GGGGT)3T	3	1,200–2,800

## Discussion

Non-leguminous nitrogen-fixing agroforestry trees are of great significance in terms of ecological and economic development. The conventional methods of propagation in tree species are difficult tasks mainly because of the long lifespan of trees, and plant tissue culture could be a beneficial approach (Martínez et al., 2019; Cano et al., 2020). The timing of explant collection plays an important role in developing a successful micropropagation protocol (Tyunin et al., 2019; Mostafa et al., 2020). The physiological status of the explant is greatly influenced by the season and hence the response of the explants during *in vitro* cultures was also affected. Furthermore, explants collected in the early summer duration (March–May) exhibit a better response than those collected in winters. Similar findings have also been reported by Seth et al. (2007) in *C. equisetifolia*, where early summer was suitable for the collection of the explants. The changes in internal hormone concentration throughout the season in the host plant might be the reason for the different responses of explant in different months of the year (Das and Pal, 2005; Sharma et al., 2005). Nutrient media compositions play an important role during *in vitro* growth of the explant by providing water and nutrients to the growing tissues (Komakech et al., 2020). As observed in this study, the MS medium was found to be the optimum nutrient medium as compared to the WPM and B5 medium for the growth of the *C. equisetifolia*. The suitability of the MS medium over WPM and B5 has also been reported by other workers, such as in *Magnolia sirindhorniae* (Cui et al., 2019), *Aspilia africana* (Okello et al., 2021), and *Azadirachta indica* (Bello et al., 2022). In contrast to our study, WPM was found as a suitable growth medium for woody tree species such as *Achyrocline satureioides* (Guariniello et al., 2018), *Quercus aliena* (Li et al., 2019), *Hildegardia populifolia* (Upadhyay et al., 2020), and *Alangium salviifolium* (Pandey et al., 2022).

Plant growth regulators interact with plant cells, tissues, and organs, and play an essential role in plant growth and development. The need for optimal concentration and a combination of the PGR can be identified experimentally. Cytokinins are responsible for promoting cell division and growth and, in addition, facilitate cell differentiation, shoot formation, and induce axillary bud growth and lateral shoot formation (Loyola-Vargas and Ochoa-Alejo, 2018; Mitrofanova, 2021). In our study, the nodal segment failed to induce axillary bud proliferation in control or MS medium devoid of any PGRs, and hence a cytokinin requirement was observed. Cytokinins, *viz*., BA, mT, Kn, and 2-iP were used in various concentrations to induce the process of direct organogenesis. As observed in this study, BA (5.0 μM) was most effective for shoot proliferation from NS of *C. equisetifolia*. The supremacy of BA among the cytokinins was also reported in *Agave salmiana* (Puente-Garza et al., 2015), *Prunus africana* (Komakech et al., 2020), and *A. africana* (Okello et al., 2021). The potential of BA to commence the division of the cell, break of bud dormancy, and development of lateral buds makes it superior to other cytokinins (Dewir et al., 2020; Rajput et al., 2020). A previous report on *C. equisetifolia* also showed the dominance of BA over other cytokinins (Seth et al., 2007). BA beyond the optimal level exhibits less and unhealthy growth in the NS. Higher concentrations of BA cause growth inhibition associated with hyperhydricity (Komakech et al., 2020), and as observed, shoot numbers are reduced at the higher concentration of BA (10.0 μM). Similar results were also observed in *Decalepis arayalpathra* and *Decalepis salicifolia* (Ahmad et al., 2018a,b).

To improve the regeneration frequency of the explant, the optimized concentration of BA (5.0 μM) was applied with auxin in a combined treatment. The synergistic effect of cytokinin and auxin is responsible for the control of main and lateral shoot formation (Bhatla, 2018; Yegorova et al., 2019). As observed, the combination of BA (5.0 μM) and NAA (0.5 μM) was found to be the optimal combination media for better growth and development. In this media, the number of shoots increased from 23.90 ± 0.40 to 32.00 ± 0.31, and similarly shoot length improved from 1.72 ± 0.07 to 3.94 ± 0.02 cm. Our findings were in accordance with a previous report on *C. equisetifolia* (Duhoux et al., 1986). The synergistic effect of BA and NAA has been demonstrated by various workers, such as in *D. salicifolia* (Ahmad et al., 2018b), *Dendrobium anosmum* (Nguyen et al., 2022), and *Diplocyclos palmatus* (Upadhyay et al., 2021). However, contrary to our results, BA in combination with IAA was found most suitable nutrient media for optimum shoot proliferation from the nodal segment of *D. arayalpathra* (Ahmad et al., 2018a) and *A. salviifolium* (Pandey et al., 2022). A regular subculture of shoots to the optimized fresh medium led to better growth in terms of multiple shoot formation. Every 3-week interval and the fourth passage of subculture gave rise to a maximum of 42.20 ± 0.82 S/E and SL of 3.45 ± 0.06 cm on an optimized medium consisting of MS + BA (5.0 μM) and NAA (0.5 μM). In addition, the shoot regeneration frequency began to decline after the fifth subculture passage. Similar results have been reported in *Cassis aungustifolia* (Parveen and Shahzad, 2011).

The rooting of *C. equisetifolia* is significantly affected by auxin type and concentration. Auxins play a significant role in the initiation and development of the root (Komakech et al., 2020). A higher frequency of rooting without basal callusing is a pre-requisite for a successful micropropagation protocol. The rooting process, especially in tree species, is considered as the most critical step and it is challenging to achieve a high rate of rooting due to several reasons, such as excessive basal callusing, phenolic production, and necrosis (Choi et al., 2003; Moyo et al., 2011). Similarly, the nutrient composition is of critical importance for healthy root induction and growth, and a half-strength medium is more efficient for rhizogenesis in woody plants (de Klerk et al., 1997). In our study, half-strength MS medium alone or in combination with the three auxins, *viz*., IAA, IBA, and NAA, was chosen for rhizogenesis. The requirement of a half-strength MS medium for root induction has been reported in several plant species, such as *Eremurus spectabilis* (Basiri et al., 2022) and *Artemisia arborescens* (Riahi et al., 2022). However, in contrast to our study, a full-strength MS medium supplemented with auxin was found suitable for root induction in the microshoots in *A. africana* (Okello et al., 2021) and *D. anosmum* (Nguyen et al., 2022). In our study, rhizogenesis was not recorded on a half-strength medium devoid of any auxin but callus formation was observed. Among the various auxins, a half-strength MS medium augmented with NAA (2.5 μM) was found to be the optimal rooting medium. The observed variation in the *C. equisetifolia* root elongation on the same hormone might be due to concentration-dependent cell elongation (Velasquez et al., 2016). Similarly, poor root growth in the presence of a higher concentration of NAA may be due to inhibitory effects caused by the higher concentration of hormones (Ivanchenko et al., 2010). The effect of auxin NAA for better rooting was also shown in various studies, such as in *Sapindus trifoliathus* (Asthana et al., 2011) and *A. africana* (Okello et al., 2021). Unlike our study, the half-strength MS medium supplemented with IBA was found to be the optimal root growth medium in *Rhododendron wattii* (Mao et al., 2018), *H. populifolia* (Upadhyay et al., 2020), *Trichilia pallida* (de Souza Prim et al., 2021), and *Eremurus spectabilis* (Basiri et al., 2022).

Acclimatization of *in vitro* derived plants with healthy shoots and roots is an important step following field survival (Hazarika et al., 2006). In our study, healthy plants of *C. equisetifolia* with well-developed shoots and roots were first transferred to a thermocol cup containing three different substrates, and among these, a combination of vermicompost + garden soil + sand (1: 2: 1) was found more suitable as 84.66% of plant survived when transferred to the field conditions after putting these hardened plants in culture room conditions for 2 weeks. No variation in morphology was recorded as compared to the mother plant. A similar substrate (soil + sand + vermicompost in the ratio of 1:1:1) was used for the acclimatization of the micropropagated plants of *Abutilon indicum* (Seth and Panigrahi, 2019).

The encapsulated somatic tissue or embryo in a gel matrix containing nutrients along with required additives are known as synthetic or artificial seeds (Redenbaugh et al., 1984; Sharma et al., 2013). The technique plays an important role for the species that produce unviable seeds or recalcitrant seeds. This method has been successfully employed in many plant species including trees for propagation and storage, such as in *Taraxacum pieninicum* (Kamińska et al., 2018), *Ansellia africana* (Bhattacharyya et al., 2018), and *Hedychium coronarium* (Behera et al., 2018). In our study, the optimized concentration of CaCl_2_ · 2H_2_O (100 mM) + Na_2_-alginate (4%) provides a clear, uniform, and ideal beads formation. Similarly, the optimized nutrient composition medium BA (5.0 μM) + NAA (0.5 μM) was found more appropriate for the conversion of encapsulated beads into the young shoot in *C. equisetifolia* in *in vitro* culture. Our results were according to the findings in *Swertia chirayita* (Kumar and Chandra, 2014), *Urginea altissima* (Baskaran et al., 2018), and *Celastrus paniculatus* (Fonseka et al., 2019). Unlike our study, CaCl_2_ · 2H_2_O (75 mM) + Na_2_-alginate (3%) was used for obtaining ideal synthetic seeds in *Rauvolfia serpentina* (Gantait et al., 2022), and CaCl_2_ · 2H_2_O (80 mM) + Na_2_-alginate (3%) in *Solanum trilobatum* (Shilpha et al., 2021). Furthermore, a 4-week period of storage at low temperature (4°C) was found to be optimal for the successful viability of synthetic seeds, and beyond it, the potential of encapsulated NS conversion into plantlets started declining. Such behavior of synthetic seeds may be due to the inhibition of tissue respiration due to the alginate and loss of moisture during a storage period of more than 4 weeks. Similar results were also reported in *Hemidesmus indicus* (Yadav et al., 2019).

Photosynthetic pigment content plays a very important role in plant health (Jiang et al., 2017). During the *in vitro* environment, controlled conditions of light and temperature are provided to the developing plantlets. Furthermore, when plants undergo *in vitro* to *ex vitro* transfer it causes physiological disablement and hence mortality can be observed at a higher rate. To deal with the above scenario, slow and cautious steps are the prerequisite and during this duration, considerable physiological changes occur in plants, and such adaptation can be more accurately understood by knowing the changes in the photosystem and its pigments (Strasser and Tsimilli-Michael, 2001). Taking into account, the pigment contents of direct regenerated plants was evaluated to determine whether they could help reduce mortality and make the micropropagation protocol more profitable in *C. equisetifolia*. In our study, the photosynthetic pigments, *viz*., chl a, b, and carotenoids decline in the first weeks of *in vitro* to *ex vitro* transfer. Imperfectly developed chloroplast and poorly developed grana as well as leaf loss in the direct regenerated plants may cause a decline in photosynthetic pigments (Siddique and Anis, 2007). Sudden environmental changes can cause photoinhibition and leaf damage, and lead to leaf fall during the initial days (Lu and Zhang, 1998; Sopher et al., 1999). New leaf formation during the second week of acclimatization resulted in an increase in photosynthetic pigments followed by a steady increase in pigment content. The increase in photosynthetic pigment content witnessed the functional health of the photosystem to various light spectra. Similar kinds of findings have also been reported in other plants, such as *D. arayalpathra* (Ahmad et al., 2018a), *D. salicifolia* (Ahmad et al., 2018b), and *Diplocyclos palmatus* (Upadhyay et al., 2021).

Scanning electron microscopy studies divulges the direct origin of shoot buds from nodal segment during *in vitro* morphogenesis. The results showed the formation of multiple shoot buds in cluster form and these shoot buds originated from the axil of each scaly leaf which later transformed into well-differentiated shoot buds. At the initial stage of shoot induction, a heavy bunch of shoot buds had formed. Similar results were also observed in *Populus deltoides* (Yadav et al., 2009), *Melia azedarach* (Vila et al., 2005), *Saccharum officinarum* (Sathish et al., 2018), and *Pinus koraiensis* (Liang et al., 2022).

Genetic fidelity assessment is essential for direct regenerated plants because they are at risk of genetic variation. In this study, RAPD and ISSR marker techniques were employed. One of the advantages of these markers is that they do not require the DNA sequence information of the sample to be known in advance, and at the same time, they are simple, cost, and time effective. Preservation of genetic similarity and uniformity in plants is essential for their growth and development (Roy, 2014). As observed in this study, no polymorphism was detected and amplified products were all monomorphic bands in *in vitro* derived plants in comparison to mother *C. equisetifolia*. The findings of this study support the maintenance of genetic similarity of direct regenerated plants with the mother plants during the *in vitro* process. The RAPD and ISSR techniques have been applied widely to examine the genetic homogeneity in various *in vitro* derived plants, such as *Magnolia sirindhorniae* (Cui et al., 2019), *Ranunculus wallichianus* (Srinivasan et al., 2021), *P. africana* (Komakech et al., 2020), *Lillium davidii* (Yang et al., 2021), and *Thunbergia coccinea* (Sultana et al., 2022).

## Conclusion

In this study, a systematic micropropagation protocol for an important multipurpose tree plant species, *C. equisetifolia*, from the nodal segment is studied. MS medium containing BA (5.0 μM) + NAA (0.5 μM) supported the maximum of 32.00 ± 0.31 S/E with SL of 3.94 ± 0.02 cm and an RP of 70% was recorded at 8 weeks of post inoculation. A rooting response of 60 % was recorded in microshoots with a root length of 3.04 ± 0.50 cm on ½ MS + NAA (2.5 μM) and 12.68 ± 0.33 roots/shoot within 6 weeks of transfer. The highest plant survival percentage of 95.10% was recorded when micropropagated plants were acclimatized in vermicompost + garden soil + sand (1:2:1). A solution with 4% of sodium alginate + 100 mM calcium chloride was found to be optimal for the formation of ideal synthetic seeds and a storage period of four weeks was found to be optimal for synthetic seed viability. The variation in photosynthetic pigment content was recorded as a consequence of plant acclimatization. The SEM study of nodal segment supported the genesis of direct shoot buds that led to the formation of true-to-type plants. RAPD and ISSR primers were used and a monomorphic banding pattern confirmed the genetic similarity of direct regenerated and mother plants. In this way, the future of this economical and environmentally important forest tree with increasing demands in forestry, paper, and wood industry can be secured. However, we suggest that additional work on micropropagation should be conducted particularly on indirect organogenesis. In addition, genetic transformation studies are also essential for the sustainable use of the *C. equisetifolia*.

## Data availability statement

The original contributions presented in the study are included in the article/Supplementary material, further inquiries can be directed to the corresponding author/s.

## Author contributions

ZA, VY, AS, AE, MR, and YD: conceptualization, writing—original draft, and revised preparation. AS and YD: supervision. YD: funding acquisition. All authors contributed to the article and approved the submitted version.

## Funding

This work was financially supported by the National Natural Science Foundation for Scholars of China (31870595) and the Priority Academic Program Development of Jiangsu Higher Education Institutions.

## Conflict of interest

The authors declare that the research was conducted in the absence of any commercial or financial relationships that could be construed as a potential conflict of interest.

## Publisher's note

All claims expressed in this article are solely those of the authors and do not necessarily represent those of their affiliated organizations, or those of the publisher, the editors and the reviewers. Any product that may be evaluated in this article, or claim that may be made by its manufacturer, is not guaranteed or endorsed by the publisher.

## Supplementary material

Supplementary Figure 1 Effect of different nutrient medium with BA (5.0 μM) + NAA (0.5 μM) (Mean ± SE).

Supplementary Figure 2 Effect of subculture passage (Mean ± SE).

Supplementary Figure 3 Effect of different planting substrate (Mean ± SE).
