# Supplementary figures and images for: Micropropagation, encapsulation, physiological, and genetic homogeneity assessment in Casuarina equisetifolia
Source: Front Plant Sci. 2022 Aug 18;13:905444. doi: 10.3389/fpls.2022.905444 (PMC9436273; doi:10.3389/fpls.2022.905444)

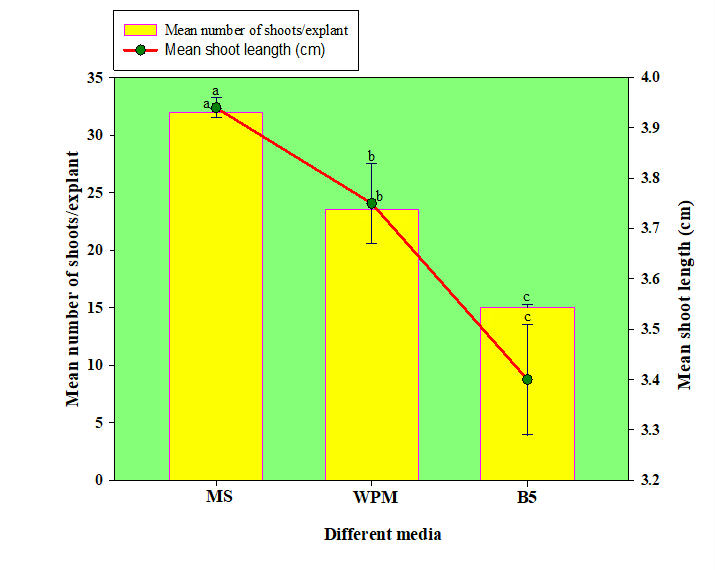

Supplement: Supplementary Figure 1 — Effect of different nutrient medium with BA (5.0 μM) + NAA (0.5 μM) (Mean ± SE). [file Image_1.TIF]

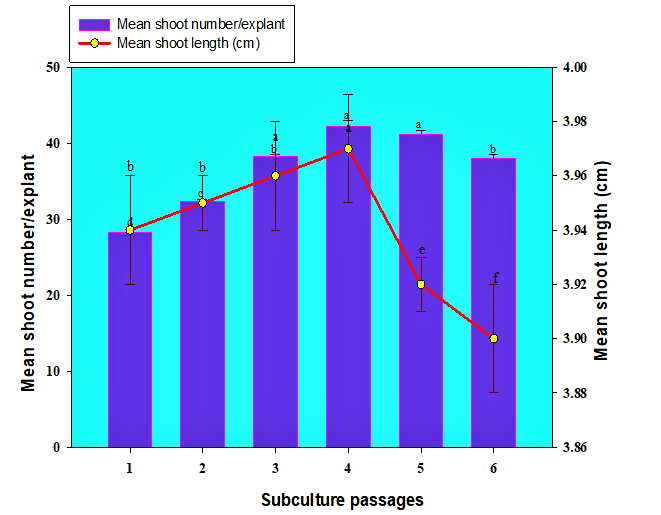

Supplement: Supplementary Figure 2 — Effect of subculture passage (Mean ± SE). [file Image_2.TIF]

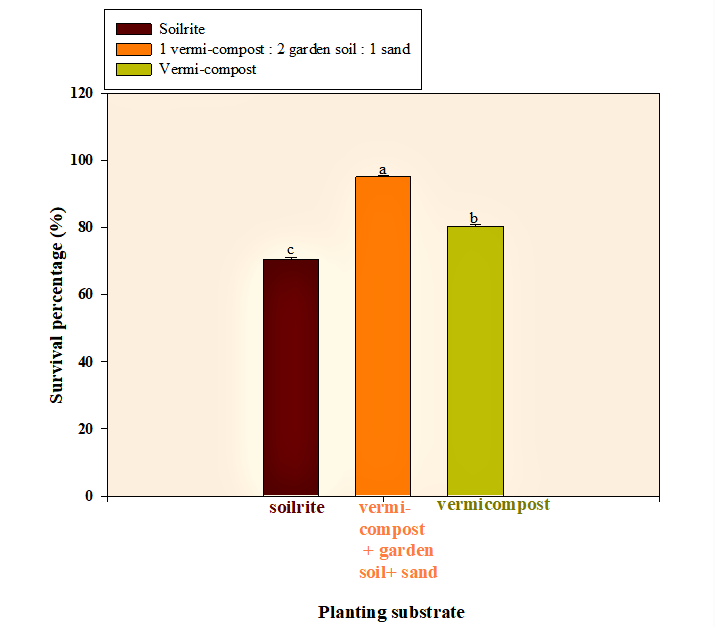

Supplement: Supplementary Figure 3 — Effect of different planting substrate (Mean ± SE). [file Image_3.TIF]
